# *Actinobacteria* from Arid and Desert Habitats: Diversity and Biological Activity

**DOI:** 10.3389/fmicb.2015.01541

**Published:** 2016-01-28

**Authors:** Fatemeh Mohammadipanah, Joachim Wink

**Affiliations:** ^1^Department of Microbial Biotechnology, School of Biology and Center of Excellence in Phylogeny of Living Organisms, College of Science, University of TehranTehran, Iran; ^2^University of Tehran Microorganisms Collection, Microbial Technology and Products Research Center, University of TehranTehran, Iran; ^3^Microbial Strain Collection, Helmholtz Centre for Infection ResearchBraunschweig, Germany

**Keywords:** *Actinobacteria*, diversity, actinomycetes, arid ecosystems, bioactive metabolites

## Abstract

The lack of new antibiotics in the pharmaceutical pipeline guides more and more researchers to leave the classical isolation procedures and to look in special niches and ecosystems. Bioprospecting of extremophilic *Actinobacteria* through mining untapped strains and avoiding resiolation of known biomolecules is among the most promising strategies for this purpose. With this approach, members of acidtolerant, alkalitolerant, psychrotolerant, thermotolerant, halotolerant and xerotolerant *Actinobacteria* have been obtained from respective habitats. Among these, little survey exists on the diversity of *Actinobacteria* in arid areas, which are often adapted to relatively high temperatures, salt concentrations, and radiation. Therefore, arid and desert habitats are special ecosystems which can be recruited for the isolation of uncommon *Actinobacteria* with new metabolic capability. At the time of this writing, members of *Streptomyces, Micromonospora, Saccharothrix, Streptosporangium, Cellulomonas, Amycolatopsis, Geodermatophilus, Lechevalieria, Nocardia*, and *Actinomadura* are reported from arid habitats. However, metagenomic data present dominant members of the communities in desiccating condition of areas with limited water availability that are not yet isolated. Furthermore, significant diverse types of polyketide synthase (PKS) and non-ribosomal peptide synthetase (NRPS) genes are detected in xerophilic and xerotolerant *Actinobacteria* and some bioactive compounds are reported from them. Rather than pharmaceutically active metabolites, molecules with protection activity against drying such as Ectoin and Hydroxyectoin with potential application in industry and agriculture have also been identified from xerophilic *Actinobacteria*. In addition, numerous biologically active small molecules are expected to be discovered from arid adapted *Actinobacteria* in the future. In the current survey, the diversity and biotechnological potential of *Actinobacteria* obtained from arid ecosystems, along with the recent work trend on Iranian arid soils, are reported.

## Introduction

The need for new bioactive structures is substantially emphasized due to the serious consequence and dynamic nature of antibiotic resistance in pathogens. Correspondingly, the need for novel bioactive compound discovery, because of their potential agricultural, pharmaceutical or industrial applications, is great (Thumar et al., 2010).

Among different resources, the privileged chemical scaffolds and metabolic potential of *Actinobacteria* have made them among the most promising bioprospecting resources (Bérdy, 2015). The rate of discovery of novel bioactive compounds has dramatically reduced in bioprospecting. As a consequence, searching for undiscovered species is imperative to address this reduction. For this purpose, either the rare genera from normal habitats or under investigated species found in unusual habitats like deserts are recommended (Harwani, 2013). Finding new actinobacterial species will presumably lead to the discovery of potentially new structural and beneficial secondary metabolites (Thumar et al., 2010). The discovery of new bioactive compounds from taxonomically unique strains of extremophilic or extremotrophic *Actinobacteria* has led to the anticipation that mining these groups could add an alternative dimension to the line of secondary metabolite resources (Thumar et al., 2010). Extremophilic and extremotolerant *Actinobacteria*, including acidtolerant and alkalitolerant, psychrotolerant and thermotolerant, halotolerant and haloalkalitolerant or xerophiles comprise the group of less investigated of this bacteria. *Actinobacteria* dwelling in deserts are capable of growing under selective conditions of pH or salinity and encompass remarkable gene clusters to produce compounds with unique antibacterial activity. However, little data is available related to the *Actinobacteria* from arid habitats, which are among the most plenteous ecosystems with regard to the occurrence of new bacterial species (Thumar et al., 2010).

By analysis of the literature data, in this review, we present the necessity of mining drought adapted *Actinobacteria*, exploring arid ecosystems for actinobacterial distribution; reporting *Actinobacteria* of arid ecosystems including studies of Iranian arid soils and bioactive metabolites of drought adapted *Actinobacteria*.

## *Actinobacteria* as the oldest and most promising resource

*Actinobacteria* are a Gram positive group often distinguished by a high mol% G+C ratio content, filamentous or non-filamentous, among which some genera produce spores (Ludwig et al., 2012). The class *Actinobacteria* comprises 5 subclasses, 10 orders, 56 families, and 286 genera (Euzeby, 2015).

*Actinobacteria* are autochthonous and often among the dominant population of their ecosystems. They have a ubiquitous distribution in the biosphere, including the extremobiosphere, and are regarded as being among the predominant components of the soil microbiota (Bull, 2011). Since the discovery of Streptomycin in 1943 (Schatz et al., 1944), the greatest number of antibiotics introduced into the market, including carbapenems (Cephalosporin), macrolides (Erythromycin), ansamycins (Rifampicin), glycopeptides (Vancomycin), and Tetracyclines (Demelocyclin), have been discovered from *Actinobacteria*. The number and diversity of biosynthetic gene clusters in their genomes, attendant with respect to the fact that only a fraction of the actinobacterial bioactive chemicals have been discovered to date, justify continuing their bioprospecting as the most promising source of novel bioactive molecules discovery.

## New source for extremophilic *Actinobacteria*

A number of environments can be considered extreme, either in terms of chemical (pH, salinity, water content) or physical parameters (temperature, pressure, radiation) (Bull, 2011). The extremophiles are evolved to thrive at or approximate to the extreme ranges of these physicochemical parameters. In contrast, a large number of microorganisms, referred to as extremotrophs, can grow but are not essentially optimized despite extreme conditions such as dilute nutrient availability that can be considered oligotroph rather than oligophile (Bull, 2011).

Members of *Actinobacteria* are recovered from a complete spectrum of extreme ecosystems. The existence of acidtolerant, alkaliphilic, psychrotolerant, thermotolerant, halotolerant, alkalitolerant, haloalkalitolerant, and xerophilous *Actinobacteria* has been reported (Lubsanova et al., 2014). Novel chemodiversity is more probable to be found in rare or recently cultivated strains. Therefore, the diversity of the extremobiosphere can resolve the challenge of rediscovery of previously known metabolites for a substantial period of time. For this reason, exploring the thriving *Actinobacteria* in extreme environments in order to obtain untapped strains is suggested. Although a few comprehensive investigations have been attempted on the bacterial diversity of arid ecosystems, the diversity of *Actinobacteria* from such environments has not been fully surveyed (Okoro et al., 2009).

## Arid habitats and existence of biogeographical barriers

Arid regions comprise the largest continental ecosystems (covering approximately 30% of all land area, of which 7% are hyper-arid) that are water-constrained. The arid areas are defined as biomes with a ratio of mean annual rainfall to mean annual evaporation of less than 0.05 and below 0.002 for extreme hyper-arid areas (Bull, 2011). The extreme desiccation condition of hyper-arid deserts is often accompanied by high temperature, nM concentrations of nutrients, low water activity, and intense radiation, while in some ecosystems, low temperature, high salinity, pH or concentrations of metals, nitrate or sulfate and inorganic oxidant anions prevail in the arid area (Bull, 2011; Koeberl et al., 2011). Among these, the availability of water and nutrients are the cardinal limiting parameters of biological activity in arid and semi-arid ecosystems (Saul-Tcherkas et al., 2013). Bacteria embedded in low water activity niches must expend rather more energy to accumulate a defined amount of water and even the most resilient bacteria usually eventuates a state of hydrobiosis when water activity is reduced to below 0.88 aw, in which cells cease to metabolize, however, remain viable (Connon et al., 2007). Bacteria that thrive in arid habitats adjust their access to water required for their physiological requirements. Most of them are adjacent to mineral soils such as quartz, halites or gypsum; through dispersal, some water trapped in these minerals can be accessed for bacterial growth (Azua-Bustos et al., 2012).

The correlation between environmental selection or stochastic processes related to the non-random dispersal of prokaryotes indicates the existence of bacterial biogeography, however, because of the exhaustive sampling required, differentiating the endemic species is difficult. Contrary to some definite similarities, arid habitats comprise diversified local physicochemical conditions that influence community structures. As a consequence, the composition of a bacterial community is the result of local environmental selection (Ragon et al., 2012) and is therefore endemic to the arid area. However, considerable population size and cell dormancy in *Actinobacteria* may have a much more determining effect on the structure of the various microbial communities, leading to different biogeographic patterns. The phylogeny-based biogeography investigation of bacteria is scarce and their functional-trait-based evaluations are even more rarely addressed (Krause et al., 2014). In addition to strain biogeography, conserved secondary metabolome enrichment patterns that are soil type–specific are also recognized in the bacterial world (Charlop-Powers et al., 2014).

Arid regions are the interface across the often vegetated semi-arid areas and the biologically unproductive hyper-arid deserts (Neilson et al., 2012). They harbor numerous unexplored xerophilic, thermophilic, halophilic and alkaliphilic *Actinobacteria* producing new bioactive metabolites. Applications of new methods can lead to the discovery of cultivable bacteria from deserts which were supposed to be sterile (Koeberl et al., 2011). The desert habitats are among the target ecosystems for the isolation of new extremophile or extremotroph strains of *Actinobacteria* which are more likely to produce new metabolites. *Actinobacteria* have exclusive tolerance to desiccation and solute stress among bacteria and they have been isolated from diverse, hostile environments such as arid and hyper-arid deserts, which are considered analogs of potential habitats on Mars (Neilson et al., 2012; Stevenson and Hallsworth, 2014). Although high levels of germination and growth at 0.5 aw is reported for *Actinobacteria*, non-halophilic species of *Actinobacteria* are unlikely to be metabolically active below 0.80 aw, however, they may be ecologically active in water constrained soil microhabitats that contain water activity above this value (Stevenson and Hallsworth, 2014). Despite the geographical extent of arid ecosystems, little is known about the bacterial populations of these habitats and their metabolic potential (Neilson et al., 2012). In this regard, few reports are available pertaining to the isolation, screening and ecological distribution of rare *Actinobacteria* from the desert ecosystem (Harwani, 2013). Additionally, habitats other than soils are also considered as new source areas with limited water availability (Azua-Bustos et al., 2012).

## Xerophilic strains isolated from arid areas

Recovered *Actinobacteria* from extremely hot and/or acidic ecosystems or habitats with severe radiation/desiccation conditions (such as deserts and other arid regions) tend to be representative of the deepest clads of *Actinobacteria* (*Acidimicrobidae, Rubrobacteridae*) (Bull, 2011). The extreme desiccating condition of deserts has been the main driving force in the evolution of the DNA repair mechanisms that has generated the resistance to ionizing radiation (UV and gamma), which is a characteristic of several desert-derived *Actinobacteria* (Makarova et al., 2001). The most resistant genera of such ecosystems are strains of *Deinococcus* and *Geodermatophilus* that tolerate up to 30 Gy of irradiation. Members of these genera have not yet been recovered from non-arid soil, even using irradiation pretreatments (Bull, 2011). Xerophilic *Actinobacteria Geodermatophilus arenarius* and *G. siccatus* were isolated from Saharan Desert sand in Chad (Harwani, 2013; Montero-Calasanz et al., 2013). Other members of the genus *Geodermatophilus* have been isolated from Negev Desert soil and from Mojave Desert soil along the California-Nevada border, together with *Actinoplanes* and *Streptomyces* strains using selective chemoattractants (Kurapova et al., 2012). The *Geodermatophilaceae* contains only two other genera of *Blastococcus* and *Modestobacter*, which thrive in the conditions of low availability of water and nutrients. *Geodermatophilus* prefers arid soils as natural habitats and out of 15 species described in this genus, at least nine species are isolated from the desert area (Euzeby, 2015), whereas *Blastococcus* and *Modestobacter* are inhabitants of rock surfaces (Montero-Calasanz et al., 2012). An actinobacterium from a desert soil in Egypt, *Citricoccus alkalitolerans*, was recognized as alkalitolerant and that its optimum growth occurs at pH 8.0–9.0 (Li et al., 2005). Novel strains of the non-sporulating actinobacterium *Mycetocola manganoxydans* that had the ability to oxidize manganese ions were isolated from the Takalima Desert (Luo et al., 2012). Members of the *Terrabacteria* genus are also characterized by adaptations to desiccation, radiation, and high salinity (Bull, 2011). Members of the genus *Streptomyces* such as *Streptomyces deserti* from the hyper-arid Atacama Desert are also reported from arid habitats (Harwani, 2013; Santhanam et al., 2013), *Streptomyces bullii* from the hyper-arid Atacama Desert (Santhanam et al., 2013) or the moderately thermophilic xerotolerant *Streptomyces* sp. 315 from Mongolian desert soil (Kurapova et al., 2012). In addition to *Streptomyces*, strains belonging to *Micromonospora, Saccharothrix, Streptosporangium*, and *Cellulomonas* were obtained from the Qinghai-Tibet Plateau (Ding et al., 2013a), while *Micromonospora, Actinomadura*, and *Nocardiopsis* were reported from soda saline soils of the ephemeral salty lakes in Buryatiya (Lubsanova et al., 2014).

Thermotolerant and thermophilic actinomycetes were found in high abundance, exceeding that of the mesophilic forms, in Mongolian desert soils. Members of *Streptomyces, Micromonospora, Actinomadura*, and *Streptosporangium* were the most widespread thermotolerant species in desert soils (Kurapova et al., 2012). Beside *Streptomyces*, members affiliated to the actinobacterial genera of *Micromonospora, Nocardia, Nocardiopsis, Saccharopolyspora*, and *Nonomuraea* have been identified from the solar salterns of the Bay of Bengal and the Arabian Sea and inland around the Sambhar Salt Lake (Jose and Jebakumar, 2012). Interestingly, it is reported that *Actinobacteria* (20.7% of desert soil and 4.6% of agricultural soil) occur at lower concentrations in farmland compared to the surrounding desert (Ding et al., 2013b). The genus *Rhodococcus* was among the dominant *Actinobacteria* in desert soil (Koeberl et al., 2011).

In particular, the resistance of halotolerant *Actinobacteria* (isolated from saline soils of arid territories) to alkaline conditions, high temperature and drought has experimentally been demonstrated. It was found that all the halotolerant strains (which were capable of growth at 5% NaCl), unlike unhalophilic strains, were able to grow on a medium that contained soda at pH 10, while non-halophilic strains do not possess such an ability. In this respect, a moderate thermophilic strain of *Streptomyces fumigatiscleroticus* 315 HE578745 that was isolated from the desert soil was experimentally shown to be xerotolerant (Lubsanova et al., 2014). The halotolerant alkaliphilic *Streptomyces aburaviensis* was isolated from the saline desert of Kutch in India that selectively inhibits the growth of Gram positive bacteria. It was able to grow at 15% w/v NaCl with slow growth at neutral pH, while optimum growth was in the range of 5–10% NaCl and at pH 9 (Thumar et al., 2010). Mesophilic *Actinobacteria* of the Mongolian desert soils ecosystem was represented by the genus *Streptomyces*, whereas thermotolerants were represented by the genera of *Micromonospora, Actinomadura*, and *Streptosporangium* (Kurapova et al., 2012).

Records of plant associated *Actinobacteria* from deserts also exist. Drought tolerant endophytic *Actinobacteria, Streptomyces coelicolor* DE07, *S. olivaceus* DE10, and *S. geysiriensis* DE27 were recovered from plants of arid and drought affected regions. These strains exhibited plant growth promotion activity and intrinsic water stress tolerance (−0.05 to −0.73 MPa) (Yandigeri et al., 2012). Some extremophilic bacteria, such as *Acidimicrobium, Rubellimicrobium*, and *Deinococcus*-*Thermus*, dramatically diminish following agricultural use. In contrary, indigenous desert bacteria can improve plant health in desert agro-ecosystems (Koeberl et al., 2011).

*Actinobacteria* from a low water activity area of Antarctica (similar to the situation in deserts) are also described. The bacterial diversity of Lake Hodgson, the Antarctic Peninsula, was recognized as 23% *Actinobacteria*, 21% *Proteobacteria*, 20.2% *Plantomycetes*, and 11.6% *Chlorofllexi* (Pearce et al., 2013), while from Antarctic Dry Valley soil *Cyanobacteria* (13%), *Actinobacteria* (26%), and *Acidobacteria* (16%) represented the majority of the identified resident bacteria (Smith et al., 2006). Culture-independent survey of multidomain bacterial diversity in the cold desert of the McKelvey Valley demonstrated that highly specialized communities colonize in distinct lithic niches occurring concomitantly within this ecosystem. Despite the relatively devoid soil, the greatest diversity was observed in endoliths and chasmoliths of sandstone. It indicated that the dominant communities are *Acidobacteria, Alphaproteobacteria*, and *Actinobacteria*. The only ubiquitous phyla in the Dry Valley zone were *Acidobacteria* and *Actinobacteria*. The overlying rock creates a favorable sub-lithic microhabitat where physical stability, desiccation buffering, water availability and irradiation protection are further provided for bactaeria (Pointing et al., 2009).

The culture independent study of *Actinobacteria* has demonstrated the dominant diversity and distribution of this phylum in arid areas. Hyper-arid soils of Yungay were shown to harbor actinobacterial OTUs (Operational Taxonomic Unit) mostly related to *Frankia* rather than the *Nitriliruptoraceae* and *Rubrobacteraceae* families that are recognized as dominant at the hyper-arid margin (Connon et al., 2007). Contrary to the fact that both regions have a sorely low level of organic substrates, higher bacterial diversity was found in the hyper-arid margin, potentially related to the mean annual rainfall and exposure to past vegetation history. Even within the hyper-arid margin, fine variations in physicochemical parameters may have a strong effect on the taxonomic diversity of actinobacterial communities (Neilson et al., 2012).

*Actinobacteria* comprised 94% of the 16S rRNA gene clones, represented the dominant group of high-powered soils of the Atacama Desert (Connon et al., 2007). The majority of isolates from this ecosystem belonged to the genera *Amycolatopsis, Lechevalieria*, and *Streptomyces* with a high incidence of non-ribosomal peptide synthase genes (Okoro et al., 2009). FISH analysis has revealed that the biomass of the metabolically active mycelial *Actinobacteria* in the prokaryotic community of Mongolian desert soils exceeded that of the unicellular *Actinobacteria* (Kurapova et al., 2012).

The overall phylum-level composition of many arid areas is shown to be dominated by *Actinobacteria*. They were shown to be the most dominant phylum (72–88%) in the case of the Atacama Desert (Crits-Christoph et al., 2013), while in other arid areas, they are among the three most abundant phyla (usually along with the Firmicutes and Proteobacteria) such as the desert soil of Aridic Calcisols in Kazakhstan (Kutovaya et al., 2015), saline–alkaline (Keshri et al., 2013), a shrub root zone of deserts (Steven et al., 2012) and high elevation desert (Lynch et al., 2014). Prevalent actinobacterial genera are not reported in almost all metagenomic studies, other than a study on the semi-arid haloalkaline ecosystem of India, in which two thirds of actinobacterial clones were recognized in the order *Rubrobacteriales* (Keshri et al., 2013).

## Biologically active metabolites reported from xerophilic *Actinobacteria*

It was hypothesized before that extremophiles can't produce secondary metabolites unless complex conditions are provided (Pettit, 2011). In contrast, now it is shown that bacteria from extreme ecosystems can produce new secondary metabolites even under regular conditions (Rateb et al., 2011). Although some antibiotic structures have been described from desert *Actinobacteria* (Table 2), reports on the natural products of *Actinobacteria* from arid environments are rare.

Bioactive molecules of the arid inhabiting *Actinobacteria* have exhibited relatively high thermal stability, bioavailability and solubility. Two new *Streptomyces* species from Atacama Desert soils (Santhanam et al., 2011, 2013) were shown to produce new ansamycin and 22-membered macrolactones with antibacterial and antitumor activity (Rateb et al., 2011). Another *Streptomyces* strain isolated from the Chilean highland soil of the Atacama Desert produces novel aminobenzoquinones which show inhibitory activity against bacteria and dermatophytic fungi (Schulz et al., 2011).

The diversity of a population comprising 52 halophilic desert actinomycetes showed the presence of strains from the *Actinopolyspora, Nocardiopsis, Saccharomonospora, Streptomonospora*, and *Saccharopolyspora* genera. Half of the strains were bioactive and harbored genes encoding polyketide synthetases and non-ribosomal peptide synthetases (NRPS). NRPS genes were widely distributed among these taxa, whereas PKS-I genes were detected in fewer genera (Meklat et al., 2011).

Endophytic *Actinobacteria* obtained from arid living plants belonging to the genera including *Streptomyces, Micromonospora, Nocardia, Nonomuraea*, and *Amycolatopsis* exhibit a high percentage of bioactivity and broad spectrum bioactivity (Huang et al., 2012). In another study, 53 *Actinobacteria* isolated from the Qinghai-Tibet Plateau were grouped into four RFLP patterns and identified as *Streptomyces, Micromonospora, Saccharothrix, Streptosporangium*, and *Cellulomonas*. Most of these strains had the potential to produce active compounds in addition to the detection of NRPS, PKS-I, and PKS-II genes (Ding et al., 2013a). Hence, the metagenomic analysis of the bioactive secondary metabolites (Schofield and Sherman, 2013; Wilson and Piel, 2013) can also be assessed in the future, in order to distinguish the chemical potential of drought adapted *Actinobacteria* and their conserved secondary metabolites biosynthetic pathways.

## Enzymes reported from xerophilic *Actinobacteria*

Two thermophilic *Rhodococcus* and *Streptosporangium* were isolated from a mud volcano in India (Ilayaraja et al., 2014). According to another report, the abundance of thermotolerant *Actinobacteria* can reach the number of mesophilic ones in deserts and volcanic regions (Zenova et al., 2009) that belonged to *Thermomonospora, Microbispora, Saccharopolyspora, Saccharomonospora*, and *Streptomyces* (Kurapova et al., 2012). A number of hydrolytic enzymes such as amylases, xylanases and cellulase from thermotolerant *Actinobacteria* can maintain their enzymatic activity, even at high temperatures (50–65°C) (Stutzenberger, 1987). A number of *Actinobacteria* like members of *Streptomyces* have been reported that grow well at 50°C (Kim et al., 1999). Thermo stable enzymes derived from such strains can be explored for potential application in industry for enzymatic digestion purposes at higher temperatures (Ilayaraja et al., 2014). Proteolytic activity of alkaliphilic, halotolerant *Actinobacteria* is also reported. Out of 42 alkaliphilic isolates, 30 isolates were reported as halotolerant alkaliphilic *Actinobacteria* with the ability to produce extracellular protease (Ara et al., 2012).

## *Actinobacteria* from arid regions of Iran and their potential biotechnological activities

**Figure 1:**
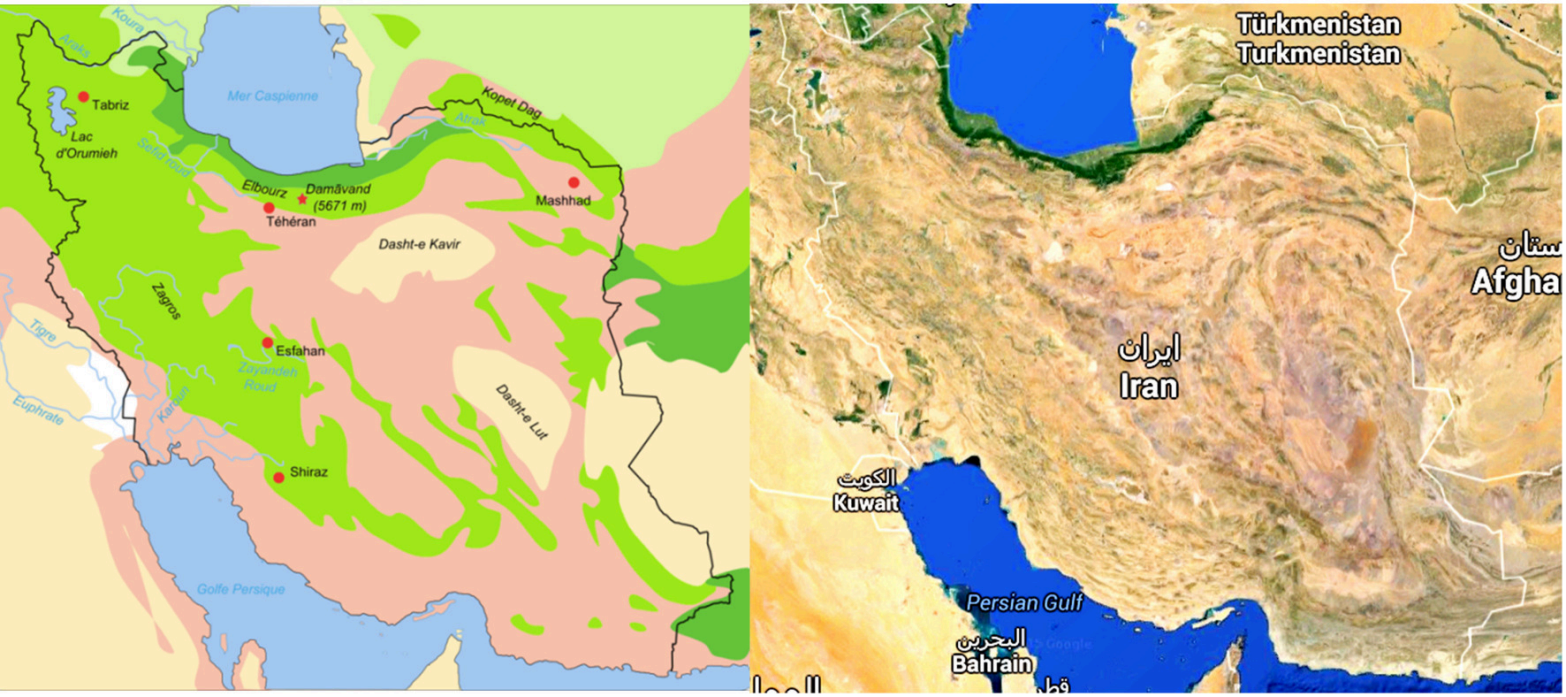
**Desert area of Iran indicated in light pink and cream in this biotope (Fabienkhan., 2006)**. 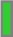, Forests and woodlands; 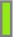, Forest steppe; 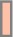, Semi-desert; 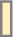, Desert lowlands; 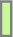, Steppe; 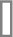, Salted alluvial marshes.

The majority of Earth's deserts have an average annual rain (AAR) of less than 400 mm per year. In turn, “true deserts” receive less than 250 mm of AAR (Azua-Bustos et al., 2012). Iran has substantial areas of arid ecotopes, including deserts (Figure 1), which are presumed to harbor xerophiles including those from the phylum *Actinobacteria*. The Plateau of Iran has two plains. Dasht-e Lut (Lut Desert) and Dasht-e Kavir (Great Salt Desert) are the main deserts of this plateau. The Great Salt Desert is about 800 km long and 320 km wide (the world's 23rd largest desert) and has mosaic-like salt plates. The Lut Desert, 480 km in length and 320 km in width (the world's 25th largest desert), is a large salt desert. It is amongst the world's driest and hottest deserts (temperatures as high as 70.7°C have been recorded) and is largely considered an abiotic zone (Mildrexler et al., 2011).

These deserts are exposed to high solar radiation, including elevated UV-B. The Lut Desert is the hottest place on earth and the Great Salt Desert contains unusually high concentrations of salt deposits. It has been assumed that the Lut Desert represents the dry and high temperature limit of bacterial metabolism and very low or zero viable bacterial content is predicted for the Lut Desert, which should be confirmed by the inability to recover amplifiable DNA from this region in future works.

Although studies on the world's deserts are increasing, information on the diversity of *Actinobacteria* in the arid areas of Iran is scarce. Up until now, only four new species of *Actinobacteria*, which belonged to the genera *Nocardiopsis, Kribbella*, and *Promicromonospora*, have been reported from the semi-arid soil of Iran (Hamedi et al., 2011; Mohammadipanah et al., 2013, 2014). Adaptation of these strains to the extreme environmental conditions of low relative humidity, high salt concentration (including toxic ions) or high UV radiation, etc. can confer on them different metabolic potential, which may lead to the exploration of new bioactive molecules.

The diverse ecological habitats of the deserts in Iran predict diverse actinobacterial species in these ecological niches. However, the ecological habitat of Iran's deserts is underexplored and yet to be investigated for their actinobacterial diversity, as reported above. Only a few actinobacterial members have been introduced from the arid areas of Iran and their secondary metabolite potential is still under investigation. Seven new species of halophilic and alkaliphilic *Actinobacteria* are described and a number of them are in the pipeline of polyphasic identification at University of Tehran. Nevertheless, their comprehensive exploitation and utilization is underinvestigated.

Application of drought adapted *Actinobacteria* in the discovery of unique bioactive compounds, enzymes, or environmental protection and sustainable agricultural application is recommended. For instance, production of the metabolite from the radiation resistant strains, halotolerant microorganisms and enzymes from thermotolerant and alkaliphilc *Actinobacteria* of these ecosystems are encouraged. Further focus on indigenous *Actinobacteria* from the deserts of Iran would increase our knowledge of their occurrence, distribution, ecology, taxonomy and biotechnological potential.

## Discussion

Diverse chemical structure, wide taxonomical spectrum, and environmental dispersal have kept *Actinobacteria* among the most reliable sources for new antibiotic discovery. Drought, extreme temperature, salinity and alkalinity and oligotrophy led to the isolation of halophilic, alkaliphilic, thermophilic and radiation resistant *Actinobacteria* (Pan et al., 2010). Designing competent culture conditions for extreme environments is an approach to exploit more biodiversity from such habitats. Additionally, their extensive stress tolerance makes them more amenable to biotechnological applications (Ding et al., 2013a).

*Actinobacteria* from Salar and extreme hyper-arid soils have been isolated using the application of pretreatment or selective media and members of at least 12 genera have been reported. A remarkable proportion of these isolates belonged to rare genera and represented new species. Members of the *Streptomyces* genus are reported as being remarkably abundant in Atacama Desert habitats and a distinguished clade with a widespread range of antibacterials and differing modes of action has been isolated from this desert. These *Streptomyces* strains are in fact Salar adapted ecovars (Bull and Asenjo, 2013). By application of a confined type of isolation media, strains of genera, including *Nocardia, Microlunatus, Prauserella*, and *Streptomyces* were recovered, and around 50% of them produced carotenoids with antibacterial activity, even against Gram negative bacteria (Namitha and Neqi, 2010). Aminobenzoquinones (rare combinations of benzoquinones and a range of amino acids) are reported from *Streptomyces* strains isolated from the Salar de Tara. Despite the poor antibacterial and antifungal activities of abenquines, inhibitory activity against type 4 phosphodiesterase (PDE4b) was revealed for them, suggesting that they can be further assessed for their anti-inflammatory activities (Schulz et al., 2011; Bull and Asenjo, 2013). The bacterial communities of another high altitude Salar, the Salar de Huasco (3800 m) were reported to be prevailed by members of *Alphaproteobacteria*, specifically, the *Roseobacter* clade. Radiation protection, sulfur cycling or regulation of the community structure by quorum sensing and the production of bioactive compounds are among the ecological functions of these bacteria in such ecosystems (Bull and Asenjo, 2013).

It is postulated that extremotolerants may have larger genetic and metabolic plasticity. Drought and radiation are life-controlling determinants, while habitat availability, temperature, pH and toxicants (high localized concentrations of elements such as arsenic) are among other principal determinants. Avoidance strategies to desiccation and intense radiation are evolved by bacteria, such as growth niche (hypo- and endo-lithic), extracellular polymer synthesis and pigmentation that protect the cell during epilithic colonization. Melanins are produced by many *Actinobacteria* thriving in extreme hyper-arid ecosystems. The dominant abundance of bacteria in a hyper-saline habitat was detected at a depth of about 2 m where water films had been formed by the aid of halite, nitrate and perchlorate salts. These suggest enough evidence to show that microorganisms in desert environments can be metabolically functional and not necessarily dormant or non-functional cells (Bull and Asenjo, 2013).

In desert habitats, the availability of water and organic substrates are among the main parameters limiting the ability of bacteria to maintain their metabolic functions (Saul-Tcherkas et al., 2013). Organic substrates can originate from the chemical profile of the plant root exudates, which induces variability in the associated bacterial composition of the arid soil (Saul-Tcherkas et al., 2013). These ecophysiological conformities such as excretion of chemicals, support an allelopathic habitat by altering the levels of organic matter and soil moisture. The significant differences in plant ecophysiological allelopathic adaptation reflect a strong influence on the soil bacterial community composition.

## Future perspective

The current focus of the natural product discovery is mainly on marine ecosystems (Bull and Asenjo, 2013), and arid habitats are underinvestigated habitats for this purpose. Microorganisms thriving in deserts are evolved to be less dependent on water. Other than the metabolic potential for pharmaceutical, environmental or agricultural purposes, diversity assessment of the desert ecosystems can advance our knowledge on actinobacterial ecology under extreme stress (Pointing et al., 2009).

There is a need for the development of new approaches and conditions to recover the actinobacterial strains from arid areas, nevertheless, in some cases *Actinobacteria* are the only bacteria that can be isolated (Okoro et al., 2009). The results obtained using metagenomic approaches to *Actinobacteria* in extreme environments has not yet been adequate to clearly indicate the dominant taxa in these habitats. Consequently, this level of data is not extensive enough to lead us toward their functional ecology in order to deduce their metabolic state of being metabolically active or dormant (Bull, 2011).

The ability of actinobacterial spores to germinate in very low available water environments (−96.4 MPa, 0.50 aw) enables their adaptation to drought conditions. Investigation of the desert soils demonstrates a high abundance of mycelial *Actinobacteria*, with actinobacterial isolates often adapted to high temperature, high salt concentration, and radiation (Kurapova et al., 2012). A broader spectrum of selective techniques used for the isolation of *Actinobacteria* from desert soils and of specific primers for molecular biological investigation will improve our knowledge of the diversity of *Actinobacteria* from the above mentioned ecosystems.

Desert habitats are especially rich in *Actinobacteria*, not necessarily extensive in taxonomic diversity (Table 1), and also in the genetic diversity of their biosynthetic pathways for synthesizing novel new secondary metabolites. Mining the natural habitats of the arid areas in Iran and designing improved procedures for selective isolation of key taxa is encouraged, as the inhabitants of the extreme areas are likely to produce new chemical entities.

**Table 1:** **Genera of the order *Actinomycetales* containing members which are resistant to the dominant physicochemical condition in arid areas other than members of *Rubrobacteraceae* and *Acidimicrobidae***.

**Suborder**	**Family**	**Genus**	**Physicochemical stress**	**References**
*Micrococcineae*	*Intrasporangiaceae*	*Terrabacteria*	Desiccation, UV-radiation, high salinity	Battistuzzi and Hedges, 2009
	*Microbacteriaceae*	*Mycetocola*	Desiccation, oligotrophic	Luo et al., 2012
	*Micrococcaceae*	*Micrococcus*	Low temperature, UV-radiation	Miteva et al., 2009
	*Microbacteriaceae*	*Microbacterium*	Oligotrophic	Miteva et al., 2009
	*Micrococcaceae*	*Citricoccus*	Desiccation	Li et al., 2005
	*Dermabacteraceae*	*Brachybacterium*	Low temperature, UV-radiation	Miteva et al., 2009
*Corynebacterineae*	*Nocardiaceae*	*Rhodococcus*	Low temperature, high radiation, pressure	Koeberl et al., 2011
	*Nocardiaceae*	*Nocardia*	Low temperature, UV-radiation	Babalola et al., 2009
	*Gordoniaceae*	*Gordonia*	Desiccation	Brandao et al., 2001
*Propionibacterineae*	*Nocardioidaceae*	*Nocardioides*	Desiccation	Tuo et al., 2015
*Pseudonocardineae*	*Pseudonocardiaceae*	*Amycolatopsis*	Desiccation	Okoro et al., 2009
	*Pseudonocardiaceae*	*Lechevalieria*	Desiccation, high salinity	Okoro et al., 2010
*Streptosporangineae*	*Thermomonosporaceae*	*Actinomadura*	High temperature	Kurapova et al., 2012
	*Streptosporangiaceae*	*Streptosporangium*	High temperature	Kurapova et al., 2012
*Frankineae*	*Geodermatophilaceae*	*Geodermatophilus*	Desiccation, gamma-radiation, UV-radiation	Harwani, 2013
	*Geodermatophilaceae*	*Modestobacter*	Desiccation, low nutrient, high radiation	Chanal et al., 2006
*Micromonosporineae*	*Micromonosporaceae*	*Micromonospora*	High temperature	Kurapova et al., 2012
*Streptomycineae*	*Streptomycetaceae*	*Streptomyces*	Low/high temperature, salinity, Desiccation, pressure	Okoro et al., 2009; Santhanam et al., 2011, 2013; Kurapova et al., 2012; Harwani, 2013

**Table 2:** **Bioactive metabolites of *Actinobacteria* isolated from arid area**.

**Producer strain**	**Source of strain**	**Compound**	**Structure**	**Bioactivity**	**References**
*Streptomyces* sp. strain DB634	Chilean highland of the Atacama Desert	Abenquines A–D	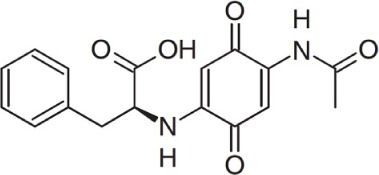	Antibacterial, antifungal and inhibition of phosphodiesterase type 4b	Schulz et al., 2011
*Streptomyces* sp. strain C34	Chilean hyper-arid Atacama Desert	Chaxalactins A-C	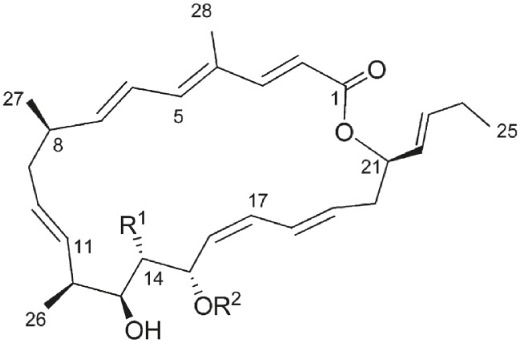	Antibacterial	Rateb et al., 2011
*Saccharothrix* sp. SA198	Saharan soil	Antibiotic A4	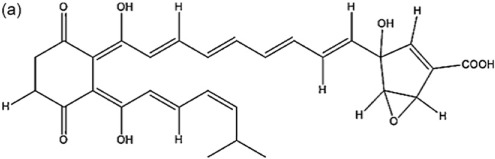	Antibacterial and antifungal	Boubetra et al., 2013
*Streptomyces* sp. TK-VL_333	Southwestern Algeria	4-(4-hydroxyphenoxy) butan-2-one	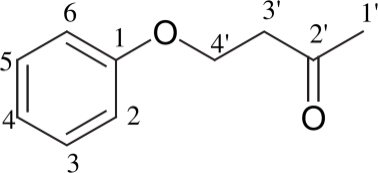	Antibacterial and antifungal	Kavitha et al., 2010
*Streptomyces* sp. TK-VL_333	Southwestern Algeria	Acetic acid-2-hydroxy-6-(3-oxo-butyl)-phenyl ester	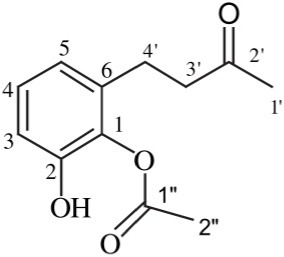	Antibacterial and antifungal	Kavitha et al., 2010

Advanced or more targeted investigations are required to more fully explore and exploit the abundance, diversity, or even the plasticity and function of actinobacterial members in desert habitats.

## Author contributions

FM wrote the manuscript and JW revised for its integrity and accuracy. FM and JW approved the final version of this manuscript and take responsibility for its contents.

### Conflict of interest statement

The authors declare that the research was conducted in the absence of any commercial or financial relationships that could be construed as a potential conflict of interest.
